# Kenny–Caffey Syndrome Type 2 (KCS2): A New Case Report and Patient Follow-Up Optimization

**DOI:** 10.3390/jcm14010118

**Published:** 2024-12-28

**Authors:** Kyriaki Hatziagapiou, Amalia Sertedaki, Vasiliki Dermentzoglou, Nataša Čurović Popović, George I. Lambrou, Louis Papageorgiou, Trias Thireou, Christina Kanaka-Gantenbein, Sophia D. Sakka

**Affiliations:** 1 Division of Endocrinology, Diabetes and Metabolism, ENDO-ERN Center for Rare Pediatric Endocrine Disorders, First Department of Pediatrics, Medical School, National and Kapodistrian University of Athens, Aghia Sophia Children’s Hospital, 11527 Athens, Greece; aserted@med.uoa.gr (A.S.); ckanaka@med.uoa.gr (C.K.-G.); sophiasakka.endo@gmail.com (S.D.S.); 2 Department of Radiology, Aghia Sofia Children’s Hospital, 11527 Athens, Greece; vasderm71@gmail.com; 3 Department of Endocrinology, Institute for Children’s Diseases, Clinical Centre of Montenegro, 81000 Podgorica, Montenegro; natasacurovic@t-com.me; 4 Choremeio Research Laboratory, First Department of Pediatrics, National and Kapodistrian University of Athens, 11527 Athens, Greece; 5 University Research Institute of Maternal and Child Health & Precision Medicine, National and Kapodistrian University of Athens, 11527 Athens, Greece; 6 Laboratory of Genetics, Department of Biotechnology, Agricultural University of Athens, 11855 Athens, Greece; lpapageorgiou@uniwa.gr (L.P.); thireou@aua.gr (T.T.); 7 Department of Biomedical Sciences, School of Health and Care Sciences, University of West Attica, 12243 Egaleo, Greece

**Keywords:** FAM111A, Kenny–Caffey syndrome type 2, gracile bone dysplasia/osteocraniostenosis, parathormone, nanophthalmos, cortical thickening, medullary stenosis, empty sella

## Abstract

**Background/Objectives:** Kenny–Caffey syndrome 2 (KCS2) is a rare cause of hypoparathyroidism, inherited in an autosomal dominant mode, resulting from pathogenic variants of the *FAM111A* gene, which is implicated in intracellular pathways regulating parathormone (PTH) synthesis and skeletal and parathyroid gland development. **Methods**: The case of a boy is reported, presenting with the characteristic and newly identified clinical, biochemical, radiological, and genetic abnormalities of KCS2. **Results**: The proband had noticeable dysmorphic features, and the closure of the anterior fontanel was delayed until the age of 4 years. Biochemical evaluation at several ages revealed persistent hypocalcemia, high normal phosphorous, and inappropriately low normal PTH. To exclude other causes of short stature, the diagnostic approach revealed low levels of IGF-1, and on CNS MRI, small pituitary gland and empty sella. Nocturnal levels of growth hormone were normal. MRI also revealed bilateral symmetrical microphthalmia and torturous optic nerves. Skeletal survey was compatible with cortical thickening and medullary stenosis of the long bones. Genomic data analysis revealed a well-known pathogenic variant of the *FAM111A* gene (c.1706G>A, p. R569H), which is linked with KCS2 or nanophthalmos. **Conclusions**: KCS2, although a rare disease, should be included in the differential diagnosis of hypoparathyroidism and short stature. Understanding the association of pathogenic variants with KCS2 phenotypic variability will allow the advancement of clinical genetics and personalized long-term follow-up and will offer insights into the role of the *FAM111A* gene in the disease pathogenesis and normal embryogenesis of implicated tissues and organs.

## 1. Introduction

Kenny–Caffey Syndrome Type 2 (KCS2) is a rare genetic condition characterized by primary hypoparathyroidism (usually transient and remittent), medullary stenosis of the long bones, proportionate short stature, ocular abnormalities, and delayed closure of the anterior fontanelle. Other clinical features include central nervous system malformations, calcification of the basal ganglia, anemia, T-cell dysfunction, minor genitourinary anomalies, microorchidism or infertility in affected males, small hands and feet, and orofacial findings [1,2,3,4,5]. It is inherited in an autosomal dominant manner, resulting from pathogenic variants of the *FAM111A* (Family with Sequence Similarity 111 Member A; FAM111 trypsin-like peptidase A) gene. *FAM111A* gene heterozygous point mutations are associated with two severe developmental syndromes: KCS2 (OMIM #127000) and Gracile bone dysplasia/Osteocraniostenosis (GCLEB/OCS, OMIM #602361) [6,7]. KCS2 was first reported in 1966 in a mother and her son who both had severe and proportionately short stature, cortical thickening, and corresponding medullary stenosis of the tubular bones. The mother documented episodes of postoperative tetany with hypocalcemia and hyperphosphatemia, delayed closure of anterior fontanelle until her late teens, and myopia. Her son also suffered from relevant episodes of hypocalcemia, with hyperphosphatemia, delayed closure of anterior fontanelle, and myopia [8,9]. Since the first reported case, only 61 cases with an identified *FAM111A* mutation have been reported globally [10,11,12]. OCS is a lethal perinatal condition characterized by gracile bones with thin diaphyses, flared and dense metaphyses, deficient calvarial mineralization, premature closure of basal cranial sutures, multiple spontaneous diaphyseal intrauterine or perinatal fractures, micromelic dwarfism, hypoparathyroidism, dysmorphic facies, eye malformations, and pulmonary and splenic hypoplasia, leading to prenatal or early postnatal death [13,14,15,16].

*FAM111A* gene is mapped on 11q12.1 and encodes a 611-amino acid chromatin-associated protein, which functions as a serine protease, with a homology of its carboxy-terminal to the chymotrypsin/trypsin subfamily of serine proteases (peptidase family S1; MEROPS database) [17,18,19]. FAM111A is expressed in all human tissues, including bones and parathyroid glands [20]. Although S1 family proteases are usually extracellular or membrane-anchored, FAM111A is located intracellularly in the nuclear and cytoplasmic cell components; however, its functions and substrates are as yet not completely understood [2,18,21]. It is suggested to have a crucial role in intracellular pathways regulating skeletal growth, parathyroid gland development, and parathyroid hormone synthesis.

In the current study, a new case report of a patient with KCS2 is presented, along with new phenotypic signs, and symptoms.

## 2. Materials and Methods

### 2.1. Patient Phenotype Analysis

The proband’s comprehensive medical family and personal history were recorded. A thorough clinical evaluation by a pediatric endocrinologist along with a medical geneticist was performed, assessing the presence of abnormalities during the patient’s initial and follow-up visits, i.e., dysmorphic features, congenital abnormalities, developmental delay, autism-like or pathological behavioral features, inborn errors of metabolism, disorders of sexual development, and ocular, ear, nose, skin, hair, teeth, cardiac, pulmonary, neuromuscular, renal, gastrointestinal, endocrinological, hematologic, immunologic, skeletal, and connective tissue disorders.

The patient’s hematological, biochemical, and endocrinological measurements and diagnostic imaging techniques at several ages were recorded and associated with clinical symptoms. In the overnight growth hormone (GH) test, blood samples for GH were drawn after sleep every 20 min (5 times), and the overnight GH surges were plotted. A peak GH response <10 mU/L is compatible with GH deficiency; a response of 10–20 mU/L suggests partial GH deficiency, whereas a response >20 mU/L is considered normal [22].

### 2.2. Dataset Collection and Filtering

A search was performed in MEDLINE, Scopus, and Google Scholar databases with the following search strings (all terms): “Kenny-Caffey syndrome, type 2” OR “KCS2” OR “Gracile bone dysplasia” OR “GCLEB” OR “osteocraniosplenic syndrome” OR “osteocraniostenosis” OR “FAM111A” OR “FAM111A protein, human” OR “autosomal dominant KCS”, with date restriction to 2013. We included case reports, case series, and review papers if they provided new patient information and a known FAM111A variant. Data were derived from the available publications related to case reports and case series, and a table was generated in which demographic and genetic data, clinical symptoms, and imaging and laboratory results were summarized (Supplementary Table S1).

### 2.3. Patient Variant Analysis

Genetic testing was performed after written informed consent from the patient’s parents. Whole Exome sequencing was undertaken employing the Human Core Exome kit (Twist Bioscience HQ, 681 Gateway Blvd, South San Francisco, CA, USA), by Sophia Genetics) on Illumina NextSeq 500. Computational analysis, including read alignment to the human genome reference sequence (GRCh37/hg19), was carried out on the SOPHiA DDM^®^ platform (Sophia Genetics, SA), and variant filtration was performed employing VarAFT V2.16 (http://varaft.eu, accessed on 10 January 2024). The pathogenic variant detected was verified by Sanger sequencing the patient’s and parents’ DNA. Informed consent for publication of the findings was obtained from the patient’s family.

## 3. Detailed Case Description

### 3.1. Patient’s Phenotype

The proband is the second child in the family, born by natural conception to non-consanguineous parents of Albanian ancestry and after an otherwise full-term, uneventful pregnancy. His birth weight and length were 3.25 kgs (SDS −0.2) and 53 cm (SDS +1.65), respectively, according to WHO Child Growth Standards [23]. Parents are clinically unaffected, and the predicted mid-parental target height (MPH) is 183 ± 4.5 cm. There is no family history of short stature, skeletal deformities, macrocephaly, or hypoparathyroidism. The patient did not exhibit prenatal growth restriction.

On the third day of life, the neonate exhibited respiratory distress attributed to birth asphyxia. On revising the boy’s personal history, several episodes of hypocalcemia were identified, which required repeated hospital admissions. An episode of neonatal seizures was recorded, which was attributed to central nervous system (CNS) infection, with concurrent hypocalcemia, hypomagnesemia, and normal blood glucose and renal function. Normocalcemia was accomplished with calcium gluconate infusion and parenteral magnesium sulfate and was maintained with oral elemental calcium and magnesium. Ophthalmological and genetic examinations during the neonatal period were negative for any abnormalities.

**Figure 1:**
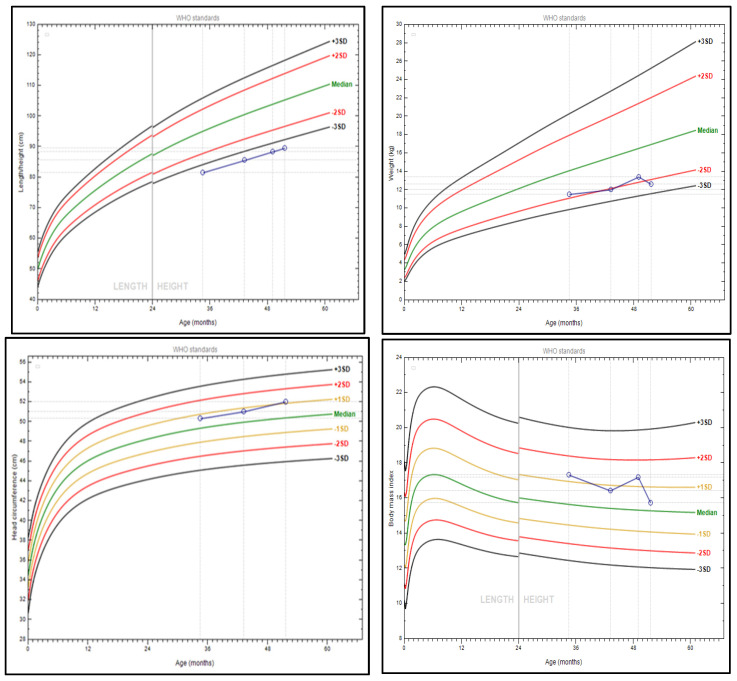
Anthropometric measurements: height (cm), weight (kg), head circumference (cm), and BMI (kg/m^2^) of the proband (WHO Child Growth Standards) [23].

At the age of 2^10/12^ years old, he was first assessed for short stature in the absence of coeliac disease, dietary problems, or any other chronic illness. His height and weight were <1st and at the 5th centile (81.5 cm −3.7 SDS, 11.5 kg −1.7 SDS, respectively), and the head circumference was at the 75th centile (50.3 cm +0.7 SDS). Poor linear growth persisted at the age of 4^3/12^ years, as his height and weight were <3rd centile (−3.7 SDS and −2.3 SDS, respectively). Head circumference remained disproportionate to the child’s height, as it was at the 86th centile (52 cm +1.1 SDS) (Figure 1). The closure of the anterior fontanel was delayed until the age of 4 years. His first tooth erupted at the age of 9 months.

Evaluation of the proband by a geneticist at the ages of 2^10/12^ and 4^1/12^ years revealed noticeable dysmorphic features, i.e., frontal bossing, macrocrania, small palpebral fissures, epicanthus, microphthalmia, blue sclerae, low set ears, long philtrum, thin upper lip, small pinched upturned nose, micrognathia, pectus excavatum, and genu valgum. The patient had proportionate limb shortening with additional circumferential skin folds. The developmental milestones were achieved at the expected ages.

**Figure 2:**
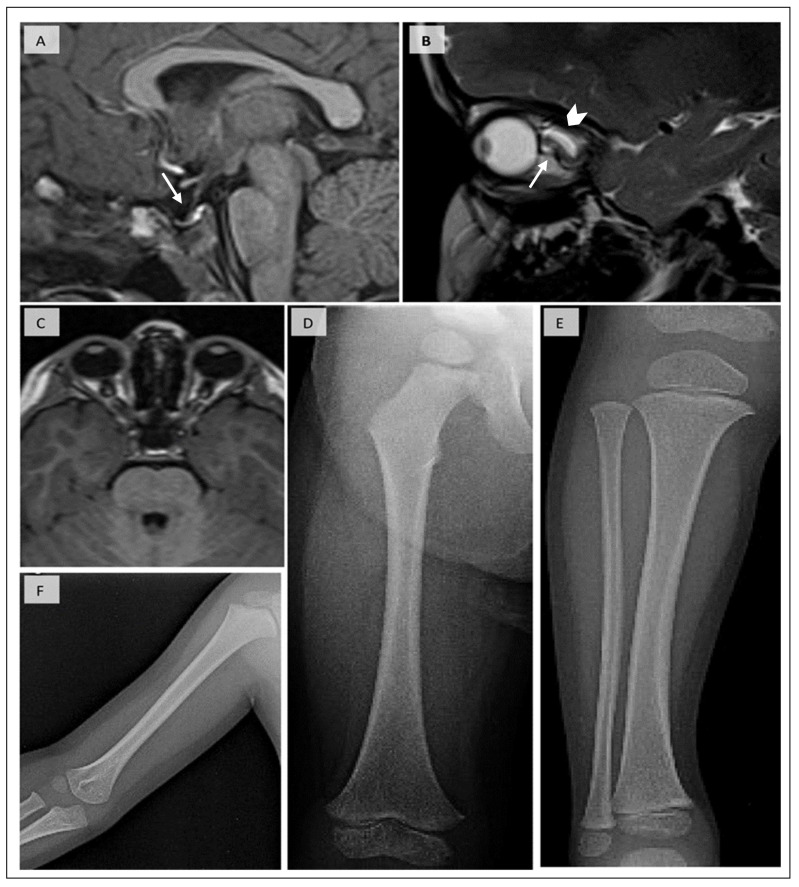
(**A**) T1-w midsagittal image shows hypoplasia of the anterior pituitary with a concave surface and almost empty sella appearance (arrow). The stalk is normal, and the posterior pituitary is orthotopic. (**B**) T2-w sagittal image demonstrates tortuosity of the optic nerve (arrow) and dilatation of the subarachnoid space surrounding its anterior portion (arrowhead). (**C**) T1-w axial image depicts bilateral symmetrical microphthalmia. X-rays of the (**D**) femur, (**E**) tibia, and (**F**) humerus show increased cortical thickness of the diaphysis, with mild narrowing of the medullary cavities. Relative flaring of the proximal tibial metaphysis.

Biochemical evaluation at several ages revealed persistent hypocalcemia, high normal phosphorous, and inappropriately low normal PTH before proper treatment initiation (Table 1). A comprehensive neurological, cardiovascular, and ophthalmological examination was negative for any pathology. Ultrasound of the upper and lower abdomen and echocardiogram were normal. To exclude other causes of short stature, routine investigations were conducted, revealing low levels of IGF-1 and, on CNS MRI, a small pituitary gland (height of 2 mm at the age of 3 years; normally, the pituitary gland height in infants and children is 6 mm), and almost empty sella appearance (Figure 2). However, nocturnal levels of growth hormone were normal (maximum GH 21.2 ng/mL). MRI also revealed bilateral symmetrical microphthalmia and torturous optic nerves. Cortisol and thyroid function tests were within normal ranges. The proband had a normal male 46, XY karyotype. His bone age (B.A.) was almost compatible with his chronological age (B.A. was calculated at 3^6/12^ years and at the age of 4 years).

A skeletal survey demonstrated cortical thickening and medullary stenosis of the long bones (humerus, ulna, radius, metacarpals, femur, tibia), flared and dense metaphyses of the proximal tibia and distal femur, and restricted growth of viscerocranium bones compared to the calvaria bones. Based on the above clinical data, serum biochemistry, and typical radiological findings, KCS2 was suspected.

### 3.2. Patient Variant Analysis

Whole exome sequencing revealed the presence of the heterozygous *FAM111A* gene (ΝΜ_001312909.2) variant c.1706G > A resulting in the substitution of Arginine for Histidine at codon 569, p.Arg569His. in the patient’s genetic profile. There was no pathogenic variant identified on *TBCΕ, AIRE, ATP7B, PTHR1,* and *GATA4* genes in WES data. Sanger sequencing of exon 6 of the *FAM111A* gene verified the presence of the variant in the patient’s DNA, and segregation analysis revealed that it is a de novo variant. *FAM111A* p.Arg569His is a well-known pathogenic variant (dbSNPID: rs587777011), which is classified as pathogenic when applying the criteria of the ACMG (American College of Medical Genetics and Genomics) and the AMP (Association for Molecular Pathology) [26]. Furthermore, this variant has been classified as pathogenic by ClinVar (RCV000050209) and has been reported in the literature multiple times as de novo in individuals with KCS2 or nanophthalmos [1,14,27,28,29,30].

### 3.3. Follow-Up

Intermittent episodes of hypocalcemia were observed during the patient’s follow-up but were not complicated by seizures and signs of hypocalcemia (e.g., Chovstek’s and Trousseau’s signs). Hypoparathyroidism was treated with oral alfacalcidol (0.5–1 mcg/day), with dose titration based on calcium and PTH levels. Alfacalcidol is as effective as calcitriol in achieving optimal calcemic control in hypoparathyroidism [31,32]. Also, dietary instructions were given to reach the daily recommended calcium intake by including at least three dairy portions in his diet. Oral elemental calcium was administered intermittently during the neonatal and infantile periods. The treatment goal is to maintain serum calcium at the lower half or just below the normal reference values to avoid hypercalciuria and to maintain PTH and 1.25(OH)_2_D levels within the optimal reference range [33,34,35,36]. During his follow-up, renal function and urinary calcium excretion were normal, whereas the ultrasound of the urinary tract was negative for nephrocalcinosis or nephrolithiasis. His ophthalmological evaluation did not reveal any complications due to the microphthalmia and calcium and vitamin D supplementation.

## 4. Discussion

**Figure 3:**
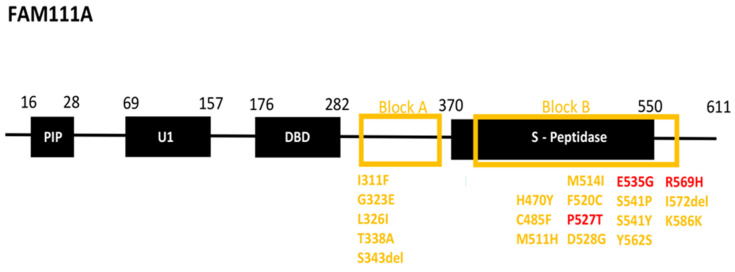
The *FAM111A* gene variants associated with KCS2 and other related diseases. Pathogenic variants that have been directly associated with KCS2 (red) and likely pathogenic variants (yellow).

FAM111A comprises four main domains: the trypsin 2-like serine protease domain at the C-terminus, two ubiquitin-like domains (UBL-1 and UBL-2), and the N-terminal PCNA (proliferating cell nuclear antigen) interacting peptide (PIP) box [20,37,38,39,40] (Figure 3). The C-terminus domain contains the chymotrypsin-like serine peptidase domain (SPD), which is highly conserved among S1 serine proteases [6,41]. The C-terminal SPD harbors the conserved catalytic triad of histidine (His), aspartate (Asp), and serine (Ser) residues (His385, Asp439, and Ser541), which, although they are located far from one another on the protein sequence, approach via protein folding to hydrolyze the peptide bonds of a substrate [18]. Although FAM111A substrates are largely unknown, it appears that long flexible peptide substrates, rather than globular proteins, preferentially access its narrow active site [42].

Τhe majority of pathogenic and likely pathogenic variants associated with the syndrome are located in two specific distinct regions in the *FAM111A* gene [40]. The first area (Block A) is demarcated between the DNA binding domain and the serine peptidase domain, and the second area (Block B) is located in the serine peptidase domain (Figure 3). Manifestation of KCS2 requires the presence of one of the well-known pathogenic variants found in the block B region, including p.P527T, p.E535G, and p.R569H [20,37,38,39,40,41,43] (Figure 3, colored red). In the proband, a well-known pathogenic variant was detected (c.1706G > A; p.R569H), associated with most KCS2 cases. The remaining well-known likely pathogenic variants are located in the block A and B regions (Figure 3, colored yellow).

Although KCS2 is inherited in an autosomal dominant pattern, there are only a few parental transmissions of the pathogenic variants. Indeed, the first case report of KCS described a mother-to-son inheritance [8,9]. When considering the most common pathogenic variant implicated in KCS2 (c.1706G>A; p.R569H), there are two cases implicating parental transmission; in the first case, the variant is maternally transmitted to a female patient, and in the latter, it is paternally transmitted to female twins [10,44]. The common phenotypic characteristics of KCS2 associated with the *FAM111A* c.1706G>A (p.R569H) variant are reviewed in Supplementary Table S1.

The main hormonal abnormality observed in KCS2 is hypoparathyroidism. Hypocalcemia may be symptomatic even from the neonatal period. It adequately responds to the administration of calcium and magnesium supplements and vitamin D analogs (alfacalcidol, calcitriol) [33,34,35,36]. If hypercalciuria is established, patients may be treated with thiazide diuretics and a low-sodium diet [36]. However, the R569H variant has been associated with the absence of hypoparathyroidism [29,45]. Generally, in hypoparathyroidism, the prevalence of CKD (chronic kidney disease) ranges from 2.5% to 41%, whereas nephrocalcinosis/nephrolithiasis is diagnosed in 19–38% of patients [34,46]. The most significant predictors of CKD are the patient’s age and duration of relative hypercalcemia, and for nephrocalcinosis, the degree of relative hypercalcemia and hyperphosphatemia, and the duration of hypocalcemia and hypercalcemia [46,47]. As hypoparathyroidism is established during infancy in KCS2, patients are in danger of renal complications due to hypocalcemia and treatment-related hypercalcemia; thus, regular monitoring of renal function, calcium and phosphate levels, and the urine calcium/creatinine ratio is important to avoid hypercalcemia and hypercalciuria by optimizing treatment protocols. Also, an annual renal ultrasound is suggested as part of KCS2 follow-up, especially when the urine calcium/creatinine ratio is suggestive of hypercalciuria [33,47].

Chronic hypoparathyroidism in KCS2 could be associated with progressive basal ganglia calcifications as early as 3 months of age and may be complicated in the future with neurological deficits, e.g., seizures, Parkinson’s disease, and mental disorders, e.g., mental retardation, anxiety, and depression [2]. The occurrence and progression of calcifications are associated with the duration of hypocalcemia and low calcium-to-phosphorus ratio [48,49]. Thus, a higher calcium/phosphorus ratio, achieved via early diagnosis and proper therapy, may prevent or reduce the progression of calcifications [48]. Also, regular neuropsychiatric assessment for secondary prevention of cognitive impairment is recommended in KCS2. Collectively, optimal monitoring of KCS2-related hypoparathyroidism to avoid long-term complications requires examining 25–hydroxyvitamin D levels every 6–12 months; serum creatinine, calcium, magnesium, and phosphorus levels every 3–12 months; and the urine calcium/creatinine ratio every 6–24 months [46].

Microorchidism and hypogonadism with increased FSH levels have been described in male patients with KCS2 and the *FAM111A* gene p.R569H pathogenic variant [50]. In these patients, testicular histology revealed Leydig cell hyperplasia, suggesting a possible role of *FAM111A* in the development and function of male genitalia [50]. Interestingly, although there are few inherited cases of *FAM111A,* no father-to-son inheritance has been described to date. In female patients, primary ovarian insufficiency and hypothalamic amenorrhea have been reported [12,28,51].

Most of the KCS2 cases, including our patient, exhibit average birth weight and length, whereas proportionate growth retardation is usually apparent postnatally (mean height SDS −5.23 ± 1.7; mean weight SDS −2.42 ± 3 for all recorded cases) [2,12,28,52]. As with other skeletal dysplasias, short stature in KCS2 is not usually associated with GH–IGF-1 axis perturbation, but rather, growth retardation is associated with a skeletal defect [53]. Indeed, in most cases, GH provocative tests are normal, and IGF-1 and IGF–BP3 levels are normal or low–normal [3,12,45,51,52,54]. The patient was diagnosed in a foreign country with an absence of GH deficiency (GHD) and exhibited normal nocturnal GH and IGF-1 levels at the age of 5 years. As GH secretion demonstrates 24 h pulsatile release, with peaks occurring at the time of slow-wave electroencephalographic rhythm, stimulating agents after overnight fasting in well-standardized protocols are preferred for diagnosing GHD, i.e., glucagon, clonidine, l–dopa, arginine, and insulin [55,56,57,58]. Although the cut-off level for the diagnosis of GHD in provocative tests has changed over time, a peak GH concentration below 10 ng/mL is universally accepted. In a child with clinical criteria, it is recommended that two failed stimulating tests (GH values < 10 ng/mL), performed on two separate days, be used in order to improve the specificity of GHD diagnosis [55,56,57,59,60]. Thus, as the reproducibility of the overnight GH secretory profile is questionable, the proband is scheduled to undergo GH stimulation tests due to severe short stature ≤0.4th centile (≤−2.7 SDS) and growth below the MPH centile [22,61].

As the patient had normal nocturnal GH secretion despite low IGF-1 levels at ages < 5 years, rGH (recombinant Growth Hormone) initiation was not considered until now. Also, as visual defects are a common complication in KCS2, we have to consider that high doses of rGH are a risk factor for the development of non-proliferative retinopathy and subsequent visual deterioration, especially in the presence of a severe refractive error, which regresses on cessation of rGH [51]. There is a case report in which a female patient was treated at the age of 3^2/12^ years with rGH 4.5–4.9 mg/m^2^/week (0.17–0.2 mg/kg/week), with an improvement in height SDS from −3.86 to −3.18 and a height velocity of 6.5 cm/year during her first year of treatment. Height velocity improved to 7.2 cm/year in the second year of rGH treatment as the dose was increased to 7.24 mg/m^2^/week (0.33 mg/kg/week) [1]. In another case, a female patient with normal IGF-1 and IGF–BP3 levels and GH stimulation tests was also started on rGH at the age of 3^6/12^ years. There was an adequate initial response during the first year, as growth velocity reached 12.3 cm/year; however, this was not sustained and treatment was altered to IGF-1 therapy after two years. However, IGF-1 was discontinued after a year due to severe headaches and poor growth response; rGH was reintroduced, with an improvement in height SDS from −5.99 at baseline to −3.38 at 12 years [45]. It is suggested that in KCS2, combined therapy of magnesium, vitamin D analogs, and rGH could be more effective in the treatment of short stature [53].

The characteristic skeletal malformation is the cortical thickening and medullary stenosis of long bones, which was also observed in our case. However, there are cases where the R569H variant is associated with a normal skeletal survey or isolated medullary stenosis of tubular bones without cortical thickening [10,12,14,29,44,45,51,62,63,64,65]. Orthopedic problems are also a significant cause of morbidity in KCS2, especially during adulthood. Significant scoliosis and hyperlordosis have been described, affecting the range of spinal movement and daily activities and causing pain or neurological deficits such as weakness or muscular atrophy [21]. Thus, early orthopedic referral and regular neurological and radiological assessment should be considered, especially during puberty or rGH treatment due to rapid linear growth and adulthood due to age-related spine degeneration.

Ocular findings are a consistent feature in most KCS2 cases [66]. In KCS2 patients with the *FAM111A* gene p.R569H pathogenic variant, the most common ocular finding is hypermetropia, whereas tortuous optic nerves, as in our proband, have never been described (to our knowledge). Nanophthalmos and microphthalmos, although uncommon ocular morbidities, have been described in KCS2 patients bearing the R569H pathogenic variant, probably due to arrested development of ocular tissues or orbital osseous defects in the early stages of embryogenesis [29,66,67,68]^.^ Patients with eyes with smaller dimensions are predisposed to sight-threatening conditions such as uveal effusion syndrome, angle closure glaucoma, retinal detachment, and chorioretinal folds [29,66,67,68].

Band keratopathy with chronic corneal degeneration due to the deposition of amorphous dust-like calcium phosphate salts in the form of microcrystalline hydroxyapatite into the superficial layers of the cornea has been observed in KCS2 patients. Interestingly, these corneal grayish-to-whitish opacities are common and more pronounced in primary and secondary hyperparathyroidism, as serum calcium supersaturation may be complicated with corneal sediment formation. However, band retinopathy has been documented in conjunction with HDR and Sanjad–Sakati syndrome, both characterized by varying degrees of hypoparathyroidism [69,70,71,72,73,74]. Band keratopathy remains undiagnosed in the early stages, leading progressively to amblyopia and sensory strabismus. Also, the accumulation of the crystals may disrupt the ocular surface, causing redness, irritation, photophobia, and recurrent corneal erosions [66,75,76,77,78,79]. Aggressive calcium and vitamin D supplementation in these patients may also play a role in band keratopathy pathogenesis; thus, ophthalmic consultation should be regularly conducted in all KCS2 patients.

KCS2 patients should be routinely screened for refractive errors, i.e., myopia, hypermetropia, or astigmatism. Pseudopapilledema is a rare complication in KCS2, secondary to the compression of the glial and nervous tissue in the region of the disc in the small scleral canal of patients with nanophthalmos or microphthalmos [66]. The compression compromises axoplasmic flow, resulting in extrusion of the drusen, or is responsible for the degeneration of the retinal ganglion cell nerve fiber axons.

The p.R569H pathogenic variant is often associated with dental problems, including failed eruption of permanent dentition, oligodontia with agenesis of at least six permanent teeth, premature loss of teeth, abnormal short roots, microdontia, enamel defects, severe dental cavities, micrognathia, and malalignment of teeth [3,80,81]. Our patient did not exhibit any dental issues until the last follow-up. The syndrome is characterized by special facial features, such as a prominent forehead, low-set ears, depressed nasal bridge, deep-set eyes, beaked nose, thin upper lip, and micrognathia. The open anterior fontanelle, even until late childhood, is a consistent feature and was also observed in our patient. However, patients with the p.R569H pathogenic variant may have a normal appearance [10,12,44,45,51,62,63,64,65].

The study patient was of normal intelligence, achieving developmental milestones at expected ages. Nevertheless, the p.R569H variant has been associated with neurodevelopment delay and intellectual disability of variable severity [10,12,44,45,51,62,63,64,65]. Other features of KCS2 associated with the p.R569H pathogenic variant are hepatitis of unknown origin and abnormal liver function tests, hypogammaglobulinemia, hypothyroidism, and sensorineural hearing loss [12,28,30]. Unique symptoms in our case are the empty sella and torturous optic nerves (as observed in CNS MRI), blue sclerae, epicanthus, pectus excavatum, and genu valgum, which, to the best of our knowledge, have not been described in other KCS2 cases.

## 5. Conclusions

The interpretation and correlation of overt manifestations with genetic variation in rare monogenic syndromes is very important in establishing the syndrome’s phenotype. Gathering genetic information based on reported case studies reveals that monogenic syndromes associated with common pathogenic variants may vary phenotypically, depending on the associated variant. The interpretation of the association of specific variants with the KCS2 phenotype will allow the advancement of clinical genetics and personalized long-term follow-up and will offer insights into the role of the *FAM111A* gene in disease pathogenesis and normal embryogenesis of implicated tissues and organs.

## Figures and Tables

**Table 1:** Serial serum and urinary markers of bone metabolism and hormonal profile.

Age	Months		Years						Normal Values [24,25]
	1	9	2^10/12^	3^1/12^	3^7/12^	4^1/12^	4^3/12^	5	
Serum calcium (mmol/L)	0.77		2.01		2.2	2.44	1.97	2.32	7 days–2 years: 2.3–2.65; 3 years–18 years: 2.25–2.7
Calcium ionized (mmol/L)			0.95	1.25	1.12	1.17	1.08	1.22	Newborn 1.05–1.37 2 months–18 years 1.2–1.38 Adult 1.05–1.3
Magnesium (mmol/L)	0.58	0.8	0.71	0.75	1.02	0.63	0.7	0.69	0–6 days: 0.48–1.05 mmol/L; 7 days–2 years: 0.65–1.05 mmol/L; 2–14 years: 0.60–0.95 mmol/L)
Phosphorus (mmol/L)	3.2		2.31	1.56	0.99	1.73	1.78	1.58	0–5 days: 1.55–2.65; 1–3 years: 1.25–2.10; 4–11 years: 1.20–1.80; 12–15 years: 0.95–1.75; 16–19 years: 0.9–1.5
Alkaline phosphatase (U/L)	393		208	239	190	260	165		<2 years: 85–235; 2–8 years: 65–210; 9–15 years: 60–300; 16–21 years: 30–200
PTH (pg/mL)	10.37	19.3	19.8	7.3		7.3	20.4	10.52	12–62
Vitamin D (ng/mL)		59.4	22.4			16.4	43	15.1	20–80
TSH (mIU/L)	1.77		6.17	5.74			3.79		0–3 days: 1–20; 3–30 days: 0.5–6.5; 1–5 months 0.5–6; 6 months–18 years: 0.5–4.5
fT4 (pmol/L)			14.7	15.4			15.4		2 weeks–20 years: 10.2–26
a–TPO IU/mL			<10						<10
a–Tg (IU/mL)			<20						<20
Cortisol (nmol/L)			281						8 am: 82.7–579.3
IGF-1 (ng/mL)			15.4				21.4	45.5	Males 0–11 months: 18–79 1 year: 20–108 2 years: 24–135 3 years: 28–148 4 years: 32–165 5 years: 37–196
IGFBP3 (mg/L)			2.07						3 years: 1.6–4.5; 4 years: 1.8–4.9

## Data Availability

The original contributions presented in this study are included in the article/Supplementary Material. Further inquiries can be directed to the corresponding author(s). The raw data supporting the conclusions of this article will be made available by the authors on request.

## Supplementary Materials

The following supporting information can be downloaded at: https://www.mdpi.com/article/10.3390/jcm14010118/s1, Table S1: KCS2 c.1706G>A (p.Arg569His) genotype-phenotype correlations [2,5,10,12,14,24,25,28,29,30,44,45,51,52,53,54,62,63,64,65].
