# Supplementary material for: Kenny–Caffey Syndrome Type 2 (KCS2): A New Case Report and Patient Follow-Up Optimization
Source: J Clin Med. 2024 Dec 28;14(1):118. doi: 10.3390/jcm14010118 (PMC11721953; doi:10.3390/jcm14010118)
Supplement: Supplementary file 1 [file jcm-14-00118-s001.zip › jcm-3382103-supplementary.pdf]

Patient	Year	Sex	Age (years)	Nationality	Parental consanguinity	Radiological abnormalities Skeletal features	FAM111A variant inheritance	Presentation of Hypoparathyroidism	Growth delay	Facial features	Dental anomalies	Ocular findings	Mental-Psychomotor Development	Other	References
**1.**	2024	F	6	N/A	No	**Prenatal ultrasound:** Shortened long bones, IUGR (intrauterine growth retardation) **Skeletal survey:** Open anterior fontanelle. Normal long bones	de novo	Normal basal serum Ca, P, Mg	**At birth:** Weight −2.31 SDS Length −2.61 SDS Head circumference (HC) 0.1 SDS **6 years:** Height −5.86 SDS Weight −6.35 SDS HC −3.1 SDS	Prominent forehead, flat nasal root	Dental caries Oligodontia Delayed tooth eruption.	High hyperopia, amblyopia, tortuous retinal vessels Microphthalmia	Normal intelligence	GH deficiency Basal IGF-I within normal levels rGH initiated at $4^{2/12}$ years	[1]
**2.**	2024	F	$5^{9/12}$	N/A	No	**Prenatal ultrasound:** Shortened long bones **Skeletal survey:** Cortical thickening and medullary stenosis of long bones. Delayed bone age Open anterior fontanelle. **Brain MRI:** Cerebral calcifications	de novo	Hyperphosphatemia Normocalcemia	**At birth:** Weight −0.9 SDS Length −0.1 SDS HC 0.1 SDS **$5^{9/12}$ years:** Height −3.99 SDS Weight −3.35 SD HC −1.28 SD	High arched palate, micrognathia	Dental caries, partial loss of dental germ.	Hypermetropia, amblyopia, pseudopapilledema. Microphthalmia			[1]
**3.**	2024	F (Twin 1)	6.5	Chinese	No	**Prenatal ultrasound:** Mild shortening of extremities IUGR. **Skeletal survey:** Open anterior fontanelle. Slender diaphyses without cortical	Paternal	Hypocalcemia on the third postnatal day	**At birth:** **Weight** −2.82 SDS **6.5 years:** Height −5.25 SDS Weight −7 SDS	Prominent forehead, depressed nasal bridge, low-set ears, micrognathia, sparse hair.	Severe dental caries	Hypermetropia, astigmatism. Microphthalmia			[1]

Patient	Year	Sex	Age (years)	Nationality	Parental consanguinity	Radiological abnormalities Skeletal features	FAM111A variant inheritance	Presentation of Hypoparathyroidism	Growth delay	Facial features	Dental anomalies	Ocular findings	Mental-Psychomotor Development	Other	References
						thickening, and medullary stenosis. Micromelia **Dual-energy X-ray absorptiometry:** Low bone mineral density									
**4.**	2024	F (Twin 2)	6.5	Chinese	No	**Prenatal ultrasound:** Shortening of extremities. **Skeletal survey:** Open anterior fontanelle. Slender diaphyses without cortical thickening, and medullary stenosis. Low bone density. Micromelia	Paternal	Normal basal serum Ca, P, Mg	**At birth:** **Weight** −3.7 SDS **6.5 years:** Height −7.91 SDS Weight − 8.59 SDS	Microcephaly Prominent forehead, open metopic ridge, depressed nasal bridge, micrognathia low-set ears, sparse hair		Hypermetropia, astigmatism, pseudopapilledema. Microphthalmia	ADHD diagnosed at 5 years of age	GH in the lower limit Liver dysfunction; abnormal inferior vena cava	[1]
**5.**	2024	M	N/A	Chinese	N/A		de novo?	Normal basal serum Ca, P	Height −4.39 SDS			Hypermetropia, astigmatism			[1]
**6.**	2024	F	0.9	Chinese	No	**Skeletal survey:** Cortical thickening and medullary stenosis of long bones. Open anterior fontanelle. **Brain CT**: Basal ganglia	de novo	Neonatal generalized convulsions due to hypocalcemia, and hypomagnesaemia. Hyperphosphatemia Low PTH	**At birth:** Weight −0.7 SDS Length 0.5 SDS HC−2.4 SDS **11 months:** Height −2.7 SDS Weight −2.1 SDS	Frontal bossing, depressed nasal bridge		Microphthalmia			[1]

Patient	Year	Sex	Age (years)	Nationality	Parental consanguinity	Radiological abnormalities Skeletal features	FAM111A variant inheritance	Presentation of Hypoparathyroidism	Growth delay	Facial features	Dental anomalies	Ocular findings	Mental-Psychomotor Development	Other	References
						calcifications at 3 months. **Kidney CT:** Increased density of renal medullary			HC−0.4 SDS						
**7.**	2024	M	4		No	**Prenatal ultrasound:** Shortening of extremities **Skeletal survey:** Open anterior fontanelle at the age of 4 years. Slender diaphyses, and medullary stenosis of tubular bones.	de novo	Normal basal serum Ca, P. Low PTH	**At birth:** Weight -0.5 SDS Length -2.1 SDS **4 years:** Height −4.44 SDS Weight −2.96 SDS				Normal neurodevelopment	Normal basal ALP, and IGF-1. Micropenis, microorchidism	[1]
**8.**	2023	M	8.75	Caucasian	N/A	Delayed bone age	de novo	Neonatal seizures due to hypocalcemia. Hyperphosphatemia, and low PTH, during the first month of life.	**At birth:** Weight -0.2 SDS Length 1.49 SDS HC -1.94 SDS				Mild intellectual disability	Hepatitis of unknown origin at 9 months. Persistent hypomagnesemia Normal IGF-1.	[2]
**9.**	2007 2023	F	11.3	Caucasian	N/A	**Antenatal ultrasound** (28 weeks' gestation): IUGR, oligohydramnios **Skeletal survey:** Long bones with	de novo	Hypocalcemia during childhood. Symptomatic hypocalcemia with normal phosphate	Disproportionate intrauterine and postnatal growth retardation **At birth:**	Frontal bossing Mid-face hypoplasia	Oligodontia	Macular oedema and visual deterioration, after rGH initiation.	Normal intelligence	17q12 loss, of parental origin. GH/IGF-1 axis: normal	[2, 3]

Patient	Year	Sex	Age (years)	Nationality	Parental consanguinity	Radiological abnormalities Skeletal features	FAM111A variant inheritance	Presentation of Hypoparathyroidism	Growth delay	Facial features	Dental anomalies	Ocular findings	Mental-Psychomotor Development	Other	References
						gracile appearance without cortical thickening or medullary stenosis. Flared metaphyses. Coxa valga Genu varum Delayed closure of the anterior fontanelle with multiple Wormian bones. J-shaped sella Abnormal mandible Disproportionate short stature **Brain MRI:** Small pituitary		levels, and low PTH in adulthood.	Weight –1.70 SDS Length–3.80 SDS, HC –2.16 SDS. **2.6 years:** Length –6.83 SDS Weight –4.37 SDS HC–3.29 SDS **Height/Length** –5.09 SDS at age 1 year-old –6.44 SDS at 2 years. –7.38 SDS at 11.3 years					basal and stimulated GH, low IGF-1, low-normal IGFBP3. rGH therapy initiated at age 2.8 years, without significant response. Mild primary ovarian insufficiency. Hypokalemia Hypomagnesemia due to renal losses Recurrent respiratory infections	
**10.**	2023	F	25	Caucasian	N/A	**Skeletal survey:** Diaphyseal medullary stenosis in femur, fibula, tibia, and	de novo	Hypocalcemia, normal serum phosphate, hypomagnese	Height -5.09 SDS Weight +0.18 SDS	Frontal bossing, hypotelorism, temporal hollowing,	Severe hypodontia Palatal enamel deficiency.	Microphthalmia	Normal intelligence	CKD stage G3bA1 (eGFR 44-65	[2]

Patient	Year	Sex	Age (years)	Nationality	Parental consanguinity	Radiological abnormalities Skeletal features	FAM111A variant inheritance	Presentation of Hypoparathyroidism	Growth delay	Facial features	Dental anomalies	Ocular findings	Mental-Psychomotor Development	Other	References
						humerus, without cortical thickening Disproportional femur shortening. **Kidney CT:** small kidneys at the age of 22 years old.		mia and decreased PTH levels.		bifid nose and vertical maxillary deficiency				mL/min/ 1.73 m^2). Hydrocephalus, complicated with seizures at the age of 11 years old. Her mother, and maternal aunt who died due to renal, and heart failure, respectively, were diagnosed with KCS2, based on clinical characteristics.	
**11.**	1983 2023	M	$2^{8/12}$	Black	N/A	**Skeletal survey:** Diaphyseal medullary stenosis, and cortical thickening in all tubular bones. Thin calvaria with absent diploic space.	de novo?	Infantile hypocalcemia complicated with brief tonic convulsions. Borderline hyperphosphatemia.	**At birth:** Weight –1.65 SDS Length –1.68 SDS HC 0.03 SDS **$2^{8/12}$ years:** Height -4.69 SDS Weight -3.39 SDS		Multiple dental carries at ages $2^{8/12}$, and 10-years old		Normal intelligence	Borderline GH secretory response in dynamic GH tests Treatment with rGh was	[2, 4]

Patient	Year	Sex	Age (years)	Nationality	Parental consanguinity	Radiological abnormalities Skeletal features	FAM111A variant inheritance	Presentation of Hypoparathyroidism	Growth delay	Facial features	Dental anomalies	Ocular findings	Mental-Psychomotor Development	Other	References
						Large fontanelles		Inappropriately low PTH. Serum Mg in the upper normal level.<br><br>At the age of $3^{8/12}$ years: Hypocalcemia, and borderline hyperphosphatemia. Undetectable PTH Hypomagnesemia	HC 0.25 SDS.					unsuccessful. Low calcitonin levels. Hypercarotenemic palms and soles.	
**12.**	2023	F	N/A	Caucasian	N/A	**Skeletal survey:** Medullary stenosis, and relative cortical thickening of tubular bones. Calvarial hyperostosis. Coxa valga bilaterally. Delayed closure of anterior fontanelle. **Brain MRI**: Bilateral flattening of posterior globes.	de novo?	Neonatal hypocalcemia complicated with seizures. Hyperphosphatemia Low serum PTH	Postnatal growth retardation, observed by 4th week of life Length -3.65 SDS	Frontal bossing	Loose dentition	Congenital anomalies of optic nerves. Bilateral optic disk swelling, decreased visual acuity, amblyopia and high hyperopia, at the age of 4 years-old.		Low IGF-1 at 2 years old. Normal GH response in dynamic tests, at the age of 4 years. rGH at the age of $6^{6/12}$ years with improvement in growth velocity and	[2]

Patient	Year	Sex	Age (years)	Nationality	Parental consanguinity	Radiological abnormalities Skeletal features	FAM111A variant inheritance	Presentation of Hypoparathyroidism	Growth delay	Facial features	Dental anomalies	Ocular findings	Mental-Psychomotor Development	Other	References
														normalization of IGF-1 levels.	
**13.**	2023	F	56	Japanese	No	**Skeletal survey:** Cortical thickening and medullary stenosis of tubular bones. Brachydactyly Disproportionate short limbs. **Brain CT:** Calcifications of the capsule	N/A	At the age of 11 years: Hypocalcemia Low serum iPTH	Postnatal growth retardation, observed at 6th month of age **11 years:** Height −4.3 SDS Weight -2.8 SDS **56 years:** Height −10.4 SDS Weight -2.70 SDS	Flat nasal root	Dental carries		Normal intelligence	Vestibular dysfunction (tinnitus, dizziness). Chronic otitis media since childhood. Sensorineural deafness, especially in the high-frequency range at the age of 49 years old. Hyperuricemia and gout, at ages 24, and 33 years old, respectively.	[5]

Patient	Year	Sex	Age (years)	Nationality	Parental consanguinity	Radiological abnormalities Skeletal features	FAM111A variant inheritance	Presentation of Hypoparathyroidism	Growth delay	Facial features	Dental anomalies	Ocular findings	Mental-Psychomotor Development	Other	References
**14.**	2022	F	7	Irish Thailand	N/A	**Skeletal survey:** Mild cortical thickening and medullary stenosis of both hands. Short fifth fingers in both hands Hypermobile joints	de novo	Normal serum calcium, phosphate and PTH.	Postnatal growth retardation **5 $^{4/12}$ years** Height -2.6 SDS Weight -1.6 SDS, HC -2.6 SDS	Mild midfacial hypoplasia Retrognathia		Maculopathy with hyper- and hypo-pigmented macular lesions in both eyes at the age of 5 years old. Subretinal fluid Moderate vascular tortuosity. Nanophthalmos.	Normal intelligence		[6]
**15.**	2022	F	18	Chinese	No	**Skeletal survey:** Cortical thickening, medullary stenosis of the long bones. Delayed closure of anterior fontanel until 5 years old. Uneven hip bone size. Micromelia **Brain CT:** Symmetrical calcifications in the cerebellar hemisphere, frontotemporal parietal lobe,	de novo	Hypocalcemia Hyperphosphatemia Hypomagnesemia Low serum iPTH	**At birth:** Weight 1.18 SDS Length -1 SDS HC 0.03 SDS **18 years** Height -5 SDS Weight -2 SDS	Microcephaly Prominent forehead Depressed nasal bridge Low-set ears Micrognathia High anterior hairline, sparse scalp hair, increased quilt hair.	Partially absent dentition	Hyperopia	Normal intelligence	Sudden onset of psychosis. Hypothyroidism Obesity Abnormal liver function Hypokalemia Hypochloremia Normal 24-hour urinary calcium. Extremely	[7]

Patient	Year	Sex	Age (years)	Nationality	Parental consanguinity	Radiological abnormalities Skeletal features	FAM111A variant inheritance	Presentation of Hypoparathyroidism	Growth delay	Facial features	Dental anomalies	Ocular findings	Mental-Psychomotor Development	Other	References
						basal ganglia, and thalamus.								irregular menstruation cycle.	
**16.**	2021	F	9	Indian	N/A	**Skeletal survey:** Cortical thickening, medullary stenosis of the long bones Absence of diploic space in the skull bones.	N/A	Hypocalcemia, presenting with seizures, at the age of 5 months, and Chvostek, and Trousseau signs, at the age of 9 years. High normal phosphorous. Inappropriately low-normal PTH. Recurrent episodes of hypocalcemia, requiring hospital admissions.	Height -4.3 SDS Weight -1.12 SDS	Large head, small palpebral fissures, long philtrum, thin upper lip, small pinched nose, elfin facies		High hypermetropia			[8]
**17.**	2020	M	12	N/A	N/A	**Skeletal survey:** Cortical thickening, medullary stenosis of the long bones. Delayed closure of anterior fontanel until 12 years. Disproportionately shorter lower limbs.	de novo	Hypocalcemia, presenting with seizures, and tetany at the age of 3-weeks, and 6 years, respectively. Hyperphosphatemia Low PTH	**12 years:** Height -4.89 SDS Weight -2.62 SDS	Deep-set eyes, narrowed palpebral fissures, prominent nose, low set ears, prominent frontal bossing	Dental caries	Microphthalmia Hyperopia in both eyes	Normal intelligence	Congenital hypothyroidism Somatotropic hypopituitarism with a poor response to rGH at the ages 6-8 years.	[9]

Patient	Year	Sex	Age (years)	Nationality	Parental consanguinity	Radiological abnormalities Skeletal features	FAM111A variant inheritance	Presentation of Hypoparathyroidism	Growth delay	Facial features	Dental anomalies	Ocular findings	Mental-Psychomotor Development	Other	References
						**Brain MRI:** Calcifications of the basal nuclei, and thalamus.								Recurrent otitis media until the age of 3 years. Bilateral hypoacusis. Cryptorchidism, micropenis Asthma	
**18.**	2020	F	10	N/A	No	**Skeletal survey:** Mild diffuse bone thinning of spine. Short metacarpal bones. Delayed bone age Hallux varus **Brain CT:** Calcifications of the basal nuclei.	de novo	Hypocalcemia Hyperphosphatemia Borderline PTH	**At birth:** Weight -0.9 SDS Height -0.98 SDS HC -1.22 SDS **10 years** Weight - 4.32 SDS Height −6.38 SDS HC -3.38 SDS	Upslanting palpebral fissures, micrognathia, thin nose		Vascular tortuosity Maculopathy High hyperopia	Delayed neuropsychomotor development Intellectual disability.	Asthma	[10]
**19.**	2020	M	18	Brazilian	No	**Skeletal survey:** Cortical thickening and medullary stenosis of bones. Frontal skull with ocular hypertelorism, mastoid cells sclerosis, craniofacial disproportion.	de novo	Neonatal hypocalcemic seizures.	**At birth:** Weight -0.1 SDS Height -0.63 SDS HC -1.15 SDS **18 years** Weight -3.89 SDS Height -4.44 SDS HC -4.97 SDS	Triangular face. Microcephaly Prominent forehead with bifrontal narrowing. Mid-face hypoplasia with broad cheeks. Narrow nasal	Partial anodontia, microdontia, dental caries. Osteosclerosis, and medullary stenosis of maxilla and jaw with calcification areas	Microphthalmia Small palpebral fissures Severe hypermetropia	Neurodevelopment delay Intellectual disability with preservation of expressive speech	Micropenis Microorchidism Hypergonadotropic hypogonadism.	[11]

Patient	Year	Sex	Age (years)	Nationality	Parental consanguinity	Radiological abnormalities Skeletal features	FAM111A variant inheritance	Presentation of Hypoparathyroidism	Growth delay	Facial features	Dental anomalies	Ocular findings	Mental-Psychomotor Development	Other	References
						Lateral skull with retrognathia and dentigerous cysts. Mesomelia Knee valgus, ankle valgus **Brain CT:** Cerebral calcifications from 3 months of age, gradually progressing to affect corpus striatum center, lateral and pulvinar nuclei of the thalamus, and dentate nucleus. At the age of 17 years calcifications affected both brain hemispheres.				base with pinched nasal tip, thin lips, tea-cup shaped ears					
**20.**	2019		10	Chinese	N/A	**Skeletal survey:** Delayed anterior fontanelle closure. Cortical thickening and medullary stenosis of tubular bones.	N/A	Low PTH at the age of 1 month.	Short stature		Enamel hypoplasia	Eye abnormalities			[12]
**21.**	2017	M	18	N/A	N/A	N/A	de novo	Without hypocalcemia	Low birth parameters Severe postnatal growth retardation		Hypodontia	Central retinopathy.	Normal psychomotor development	Mild partial GH deficiency	[13]

Patient	Year	Sex	Age (years)	Nationality	Parental consanguinity	Radiological abnormalities Skeletal features	FAM111A variant inheritance	Presentation of Hypoparathyroidism	Growth delay	Facial features	Dental anomalies	Ocular findings	Mental-Psychomotor Development	Other	References
														Markedly decreased serum IGF-I. Partial rGH resistant during 4.5 to 8.5 years old. Central hypothyroidism	
**22.**	2014	F	3	Canadian	N/A	**Antenatal ultrasound:** IUGR **Skeletal survey:** Large anterior fontanelle. Medullary stenosis of tubular bones.	Maternal	Neonatal seizures due to hypocalcemia, and hypomagnesaemia Low levels of PTH	**At birth:** Weight −2.5 SDS Length - 4 SDS HC -1 SDS **3 years:** Height −5 SDS	Frontal bossing	Dental caries.	Myopia Microphthalmia			[14]
**23.**	2014	F	25 (mother of proband 22)	Canadian	N/A	**Skeletal survey:** Osteosclerotic skull, cortical thickening and medullary narrowing of long bones	de novo	Neonatal hypocalcemic seizuress	Height - 5 SDS HC −4 SDS	Microcephaly	Oligodontia Retention of primary teeth	Myopia		Hypothyroidism Sensorineural hearing loss	[14]
**24.**	2014	F	12	N/A	No	**Skeletal survey:** Cortical thickening with mild medullary stenosis of long bones Bilateral coxa valga.	de novo	Normal serum calcium, phosphate and PTH.	**At birth:** Weight - 0.31SDS Length - 1.48SDS **12 years:** Height −5.99 SDS	Prominent forehead, flat nasal bridge, curved and upturned nose	Delayed eruption of primary teeth. Small permanent teeth. Soft enamel	Severe myopia	Normal neurodevelopment	Precocious puberty Normal GH, IGF-1, IGF-BP3 levels, and GH	[15]

Patient	Year	Sex	Age (years)	Nationality	Parental consanguinity	Radiological abnormalities Skeletal features	FAM111A variant inheritance	Presentation of Hypoparathyroidism	Growth delay	Facial features	Dental anomalies	Ocular findings	Mental-Psychomotor Development	Other	References
														stimulation tests. The patient was variably treated with rGH and IGF-1 with an overall moderate response.	
**25.**	2014	F	10	Japanese	No	**Skeletal survey:** Cortical thickening and medullary stenosis of tubular bones.<br>Delayed closure of anterior fontanelle at the age of 2 years.<br>**Brain CT**: calcification in basal ganglia.	de novo	Normal levels of phosphate, and calcium at the age of 3 months.<br>Hypocalcemia, hyperphosphatemia, and low iPTH at 1 year of age.<br>Hypomagnesaemia at the age of 3 years.	**Birth**<br>Weight 0.3 SDS<br>Length -0.4 SDS<br>**3 months**<br>Weight -1.8 SDS<br>Length -2.5 SDS<br>HC 0.2 SDS<br>**10 years:**<br>Height −4.2 SDS	Macrocephaly<br>Prominent forehead, deep-set eyes, external ears abnormalities, depressed nasal bridge and micrognathia.		Hypermetropia, pseudopapilledema	Normal intelligence	Abnormal liver function during infancy<br>Polysyndactyly<br>Anemia<br>Severe GHD	[16]
**26.**	2014	M	16	Japanese	No	**Skeletal survey:** Cortical thickening and medullary stenosis of tubular bones.<br>Delayed closure of anterior fontanelle until 9 years.	de novo	Neonatal hypocalcemic seizures.<br>Hyperphosphatemia, hypomagnesaemia, and undetectable iPTH during neonatal age.	**Birth**<br>Weight -0.4 SDS<br>Length 0.5 SDS<br>**3 years**<br>Proportionate short stature<br>Weight -2.7 SDS	Prominent forehead, deep-set eyes, depressed nasal bridge beaked nose, thin upper lip, micrognathia, anteverted nares.		Hypermetropia	Normal intelligence	Repeated acute otitis media until 2 years.<br>Severe atopic dermatitis<br>Mild GHD	[16]

Patient	Year	Sex	Age (years)	Nationality	Parental consanguinity	Radiological abnormalities Skeletal features	FAM111A variant inheritance	Presentation of Hypoparathyroidism	Growth delay	Facial features	Dental anomalies	Ocular findings	Mental-Psychomotor Development	Other	References
									Height -4.4 SDS<br>HC -1.5 SDS<br>**16 years**<br>Height −8.2 SDS						
**27.**	2014	F	22	Japanese	No	**Skeletal survey:** Cortical thickening and medullary stenosis of tubular bones. Delayed closure of anterior fontanelle until 5 years.<br>**Brain CT**: Basal ganglia calcifications.	de novo	Neonatal hypocalcemic seizures. Hyperphosphatemia, hypomagnesaemia, and undetectable iPTH.	**5 years**<br>Proportionate short stature<br>Weight -2.2 SDS<br>Height -5.3 SDS<br>**22 years**<br>Height −4.5 SDS	Prominent forehead, deep-set eyes, beaked nose, thin upper lip, micrognathia.		Hypermetropia	Normal intelligence	Hypothalamic amenorrhea	[16]
**28.**	2014	M	38	Japanese	No	**Skeletal survey:** Cortical thickening and medullary stenosis of tubular bones. Delayed closure of anterior fontanelle until 12 years.<br>**Brain CT**: Basal ganglia calcifications	de novo	Neonatal seizures due to hypocalcemia, and hypomagnesaemia. Hypocalcemic seizures at the age of 4 years. Undetectable iPTH, hyperphosphatemia, and hypomagnesaemia at the age of 4 years.	**Birth**<br>Weight -0.3 SDS<br>Length -1.4 SDS<br>**12 years**<br>Proportionate short stature<br>Weight -3.3 SDS<br>Height -6.3 SDS<br>**38 years**<br>Height −5.3 SDS	Prominent forehead, deep-set eyes, beaked nose, thin upper lip, micrognathia.		Hypermetropia amblyopia, pseudopapilledema	Normal intelligence	Repeated acute otitis media during infancy. Empyema and bacterial meningitis at 4 years. Hypogammaglobulinemia	[16]

Patient	Year	Sex	Age (years)	Nationality	Parental consanguinity	Radiological abnormalities Skeletal features	FAM111A variant inheritance	Presentation of Hypoparathyroidism	Growth delay	Facial features	Dental anomalies	Ocular findings	Mental-Psychomotor Development	Other	References
**29.**	2013	F	40	Swiss	N/A	**Skeletal survey:** Basal craniosynostosis V-shaped orbital roofs	de novo	Hypocalcemia	Height −6 SDS	Triangular face Frontal bossing	Defective dentition, premature shedding	Hypermetropia, cataracts Microphthalmia		Hypoacusis, high-pitched voice.	[17]
**30.**	2013	M	17	Indian	N/A	**Skeletal survey:** Cortical thickening and medullary stenosis of tubular bones.	N/A	Hypocalcemia	Height −6 SDS		Defective dentition	Hypermetropia			[17]
**31.**	2013	M	10	German	N/A		N/A	N/A	Height −7 SDS			Hypermetropia			[17]
**32.**	2013	F	0.6	Italian	N/A		de novo	Hypocalcemia	Height -2 SDS at birth. Height -3 SDS at 6 months.						[17]

Supplementary Table S1. KCS2 c.1706G>A (p.Arg569His) genotype-phenotype correlations

Consanguinity refers to the reproductive relationship between individuals who are descendants of at least one common ancestor; thus, being first-, second-, or third-degree relatives. Postnatal growth retardation was defined as short stature of ≤−2 SD, and intrauterine growth retardation was defined as low birth weight of ≤−2 SD. In disproportionate short stature is defined as Sitting Height/Height (SH/H) SDS > +2, indicating appendicular growth impairment [18]. Delayed closure of the anterior fontanelle was defined as a still patent anterior fontanelle at the age of 26 months. Intellectual disability is a condition characterized by significant limitations in both intellectual functioning (learning, problem solving, reasoning, etc) and adaptive behavior (conceptual, social, and practical skills). CKD was defined as the presence of kidney damage or an estimated glomerular filtration rate (eGFR) less than 60 ml/min/m^2 or albuminuria of >30 mg/day persisting for > 3 months.

When considering laboratory results we considered the reference ranges, as described by Mosby's Diagnostic and Laboratory Test Reference, and Nelson Pediatrics[19, 20]. Hypocalcemia was defined as serum total calcium values below the normal lower level for a particular age group (cord blood: 2.25-2.88 mmol/L; newborn 3-24 hours: 2.3-2.65 mmol/L, 24-48 hours: 1.75-3.00 mmol/L, 4-7 days: 2.25-2.73 mmol/L; 7 days-2 years: 2.3-2.65 mmol/L; child 3 years-18 years: 2.25-2.7 mmol/L, ≥18 years: 2.10-2.55 mmol/L). Hypomagnesemia was defined as serum magnesium levels below the lower reference limit (0-6 days: 0.48-1.05 mmol/L; 7 days-2 years: 0.65-1.05 mmol/L; 2-14 years: 0.60-0.95 mmol/L). Hyperphosphatemia was considered when phosphorus serum levels

were above the upper limit of normal (0-5 days: 1.55-2.65 mmol/L; 1-3 years: 1.25-2.10 mmol/L; 4-11 years: 1.20-1.80 mmol/L; 12-15 years: 0.95-1.75 mmol/L; 16-19 years:0.90-1.50 mmol/L). Hypokalemia was defined as serum potassium levels below the lower reference limit (newborn: 3.9-5.9 mEq/L; infant: 4.1-5.3 mEq/L; child: 3.4-4.7mEq/L; adult: 3.5-5.0 mEq/L). Hypernatremia was considered as sodium levels higher than the upper normal range (newborn: 134-144 mEq/L; infant: 134-150 mEq/L; child: 136-145 mEq/L; adult: 136-145 mEq/L). Hypochloremia was defined as serum chloride below the reference range (premature infant: 95-110mEq/L; newborn: 96-106mEq/L; child: 90-110 mEq/L). Hypoglycemia was defined as fasting serum glucose below the lower limit for a particular age group (cord: 45-96 mg/dL; premature infant: 20-60 mg/dL; neonate: 30-60 mg/dL; infant: 40-90 mg/dL; 2 years to adulthood:70-110 mg/dL). High alkaline phosphatase (ALP) was defined as levels above the upper limit of normal (< 2 years: 85-235 U/L; 2-8 years: 65-210 U/L; 9-15 years: 60-300 U/L; 16-21 years: 30-200 U/L; adult: 30-120 U/L). High aspartate aminotransferase (AST; glutamic-oxaloacetic transaminase SGOT) was defined as levels above the upper limit of normal (0-5 days: 35-140 U/L; < 3 years: 15-60 U/L; 3-6 years: 15-50 U/L; 6-12 years: 10-50 units/L; 12-18 years: 10-40 units/L; adult: 0-35 units/L). High alanine aminotransferase (ALT; glutamic- A pyruvic transaminase SGPT) was defined as levels above the upper limit of normal (infant: 8-72 U/L; child/adult: 4-36 U/L). Hypercalciuria was defined as a ratio of urine calcium to urine creatinine above normal range for a particular age group (1 -12 months: 0.03-0.81 mg/mg creatinine; 12 -24 months: 0.03-0.56 mg/mg creatinine; 2-3 years: 0.02-0.50 mg/mg creatinine; 3 -5 years: 0.02-0.41 mg/mg creatinine; 5 - 7 years: 0.01-0.30 mg/mg creatinine; 7 -10 years: 0.01-0.25 mg/mg creatinine; 10 -18 years: 0.01-0.24 mg/mg creatinine; > 18 years: 0.05-0.27 mg/mg creatinine). Hypermagnesiuria was defined as a ratio of urine magnesium to urine creatinine above normal range for a particular age group (1 -12 months: 0.10-0.48 mg/mg creatinine; 12 -24 months: 0.09-0.37 mg/mg creatinine; 2-3 years: 0.07-0.34 mg/mg creatinine; 3 -5 years: 0.07-0.29 mg/mg creatinine; 5 - 7 years: 0.06-0.21 mg/mg creatinine; 7 -10 years: 0.05-0.18 mg/mg creatinine; 10 -14 years: 0.05-0.15 mg/mg creatinine; 14 -18 years: 0.05-0.13 mg/mg creatinine; > 18 years-83 years: 0.04-0.12 mg/mg creatinine).

Hypoparathyroidism was defined as serum PTH levels below the lower limit of the normal references of the intact PTH (iPTH) 10-65 pg/mL. Low calcitonin (CT) was considered as levels ≤ 19 ng/L, ≤ 14 ng/L for males, and females respectively. Hypothyroidism was defined as free thyroxine (FT4) below the lower limit of normal range for a particular age group (0-4 days: 2-6 ng/Dl; 2 weeks-20 years: 0.8-2 ng/dL; adult: 0.8-2.8 ng/dL) and/or as thyroid stimulating hormone (TSH) higher than the upper normal range (cord: 3-12 µIU/L; 0-3 days: 1-20 µIU/L; 3-30 days: 0.5-6.5 µIU/L ;1-5 months: 0.5-6 µIU/L; 6 months-18 years: 0.5-4.5 µIU/L; adult: 2-10mU/L). Growth hormone (GH) deficiency (GHD) was defined when GH stimulation test using peak values of <10 ng/mL. Insulin-like growth factor 1 (IGF-1) deficiency was assumed when values were ≤ −2 SD (or ≤3rd percentile) for specific sex, and Tanner stage. Reference values for IGF-1 are for males 0-11 months: 18-79 ng/mL; 1 year: 20-108 ng/mL; 2 years: 24-135 ng/mL; 3 years: 28-148 ng/mL; 4 years:32-165 ng/mL; 5 years: 37-196 ng/mL; 6 years: 43-229 ng/mL; 7 years: 50-243 ng/mL; 8 years: 59-275 ng/mL; 9 years: 67-315 ng/mL; 10 years: 75-366 ng/mL; 11 years: 82-423 ng/mL; 12 years: 87-519 ng/mL; 13 years: 101-620 ng/mL; 14 years: 123-701 ng/mL; 15 years: 161-760 ng/mL; 16 years: 171-748 ng/mL; 17 years: 161-635 ng/mL; 18 years: 145-506 ng/mL; 19 years: 122-435 ng/mL; 20 years: 116-410 ng/mL. For females reference values of IGF-1 are 0-11 months: 14-106 ng/mL; 1 year: 23-136 ng/mL; 2 years: 30-163 ng/mL; 3 years: 34-192 ng/mL; 4 years: 38-217 ng/mL; 5 years: 46-243 ng/mL; 6 years: 56-268 ng/mL; 7 years: 64-288 ng/mL; 8 years: 74-337

ng/mL; 9 years: 81-405 ng/mL; 10 years: 85-526 ng/mL; 11 years: 91-610 ng/mL; 12 years: 110-656 ng/mL; 13 years: 150-678 ng/mL; 14 years: 174-656 ng/mL; 15 years: 156-586 ng/mL; 16 years: 140-517 ng/mL; 17 years: 130-471 ng/mL; 18 years: 117-430 ng/mL; 19 years: 113-408 ng/mL; 20 years: 108-384 ng/mL. Reference values for IGF-BP3 (IGF-binding protein 3) are for males are 0-11 months: 1.1-3.2 mg/L; 1 year: 1.3 - 3.6 mg/L; 2 years 1.5 - 4.1 mg/L; 3 years: 1.6 - 4.5 mg/L; 4 years: 1.8 - 4.9 mg/L; 5 years: 1.9 – 5.2 mg/L; 6 years: 2 - 5.4 mg/L; 7 years: 2.1 - 5.5 mg/L; 8 years: 2.2 - 5.6 mg/L; 9 years: 2.2 - 5.7 mg/L; 10 years: 2.3 - 5.8 mg/L; 11 years: 2.4 - 6 mg/L; 12 years: 2.5 - 6.1 mg/L; 13 years: 2.5 - 6.2 mg/L; 14-16 years: 2.6 - 6.3 mg/L; and 17-19 years: 2.7 - 6.3 mg/L, and for females are 0-11 months: 1.1-3.3 mg/L; 1 year: 1.2 - 3.7 mg/L; 2 years 1.4 - 4.2 mg/L; 3 years: 1.6 - 4.6 mg/L; 4 years: 1.7 - 4.9 mg/L; 5 years: 1.9 – 5.2 mg/L; 6 years: 1.9 - 5.4 mg/L; 7 years: 2 - 5.5 mg/L; 8 years: 2.1 - 5.6 mg/L; 9 years: 2.2 - 5.8 mg/L; 10 years: 2.3 - 5.9 mg/L; 11 years: 2.4 – 6.1 mg/L; 12 years: 2.4 - 6.2 mg/L; 13 years: 2.5 - 6.3 mg/L; 14-15 years: 2.6 - 6.4 mg/L; 16-19 years: 2.7 - 6.5 mg/L. Reference values for LH (Luteinizing Hormone) are for males 1 -12 months: ≤0.4 IU/L; 1-6 years: ≤1.3 IU/L; 6-11 years: ≤1.4 IU/L; 11-14 years: 0.1-7.8 IU/L; 14-18 years: 1.3-9.8 IU/L; >18 years: 1.3-9.6 IU/L, and females 1 -12 months: ≤0.4 IU/L; 1-6 years: ≤0.5 IU/L; 6-11 years: ≤3.1 IU/L; 11--14 years: 11.9 IU/L; 14-18 years: 0.5-41.7 IU/L; >18 years: 1.3-9.6 IU/L (Premenopausal: follicular: 1.9-14.6 IU/L; midcycle: 12.2-118.0 IU/; luteal: 0.7-12.9 IU/L). FSH (Follicle-Stimulating Hormone) reference values , according to Tanner stages are for males Stage l: <1.5 IU/L; Stage ll: <3.0 IU/L; Stage lll: 0.4-6.2 IU/L; Stage lV: 0.6-5.1 IU/L, and Stage V: 0.8-7.2 IU/L, and for females Stage l: 0.6-4.1 IU/L; Stage ll: 0.3-5.8 IU/L; Stage lll: 0.1-7.2 IU/L; Stage lV: 0.3-7.0 IU/L; Stage V: 0.4-8.6 IU/L (Premenopausal: follicular: 2.9-14.6 IU/L; midcycle: 4.7-23.2 IU/L; luteal: 1.4-8.9 IU/L). Total testosterone reference values are for males according to age: 0-5 months: 75-400 ng/dL; 6 months-9 years: < 7-20 ng/dL; 10-11 years: < 7-130 ng/dL; 12-13 years: < 7-800 ng/dL; 14 years: < 7-1200 ng/dL; 15-16 years: 100-1200 ng/dL; 17-18 years: 300-1200 ng/dL; ≥19 years: 240-950 ng/dL, and according to Tanner stages: Tanner I (prepubertal): <7-20 ng/dL; Tanner II: 8-66 ng/dL; Tanner III: 26-800 ng/dL; Tanner IV: 85-1,200 ng/dL; Tanner V: 300-950 ng/dL. Total testosterone reference values are for females according to age: 0-5 months: 20-80 ng/dL; 6 months-9 years: < 7-20 ng/dL; 10-11 years: < 7-44 ng/dL; 12-16 years: < 7-75 ng/dL; 17-18 years: 20-75 ng/dL; ≥ 19 years: 8-60 ng/dL, and according to Tanner stages: Tanner I (prepubertal):< 7-20 ng/dL; Tanner II: < 7-47 ng/dL; Tanner III: 17-75 ng/dL; Tanner IV: 20-75 ng/dL; Tanner V: 12-60 ng/dL.

Abbreviations; Arginine-Arg; Histidine-His; IUGR intrauterine growth retardation; GH Growth Hormone; rGH recombinant growth hormone; CKD Chronic kidney disease; eGFR estimated glomerular filtration rate; ALP alkaline phosphatase; AST/ SGOT aspartate aminotransferase/ glutamic-oxaloacetic transaminase; ALT/ SGPT alanine aminotransferase/ glutamic- A pyruvic transaminase; CT calcitonin; PTH parathormone; FT4 free thyroxine; TSH thyroid stimulating hormone; IGF-1 Insulin-like growth factor 1; LH Luteinizing Hormone; FSH Follicle-Stimulating Hormone

1. Chen, X. and C. Zou, *Further delineation of phenotype and genotype of Kenny-Caffey syndrome type 2 (phenotype and genotype of KCS type 2).* Mol Genet Genomic Med, 2024. **12**(4): p. e2433.
2. Schigt, H., et al., *Expanding the Phenotypic Spectrum of Kenny-Caffey Syndrome.* J Clin Endocrinol Metab, 2023. **108**(9): p. e754-e768.
3. Paterson, W.F., et al., *Deterioration of visual acuity associated with growth hormone therapy in a child with extreme short stature and high hypermetropia.* Horm Res, 2007. **67**(2): p. 67-72.
4. Lee, W.K., A. Vargas, J. Barnes, and A.W. Root, *The Kenny-Caffey syndrome: growth retardation and hypocalcemia in a young boy.* Am J Med Genet, 1983. **14**(4): p. 773-82.
5. Ohmachi, Y., et al., *Case report: Late middle-aged features of FAM111A variant, Kenny–Caffey syndrome type 2-suggestive symptoms during a long follow-up.* 2023. **13**.
6. Lang, E., et al., *Genotype-phenotype spectrum in isolated and syndromic nanophthalmos.* Acta Ophthalmol, 2021. **99**(4): p. e594-e607.
7. Yuan, N., et al., *Clinical and genetic features of Kenny-Caffey syndrome type 2 with multiple electrolyte disturbances: A case report.* World J Clin Cases, 2023. **11**(10): p. 2290-2300.
8. Yerawar, C., A. Kabde, and P. Deokar, *Kenny-Caffey syndrome type 2.* Qjm, 2021. **114**(4): p. 267-269.
9. Kaleta, D., P. Zapolnik, A. Mazur, and A. Pyrkosz, *A rare cause of short stature: Kenny-Caffey syndrome type 2 – a case report and literature review.* Pediatria Polska - Polish Journal of Paediatrics, 2020. **95**(4): p. 249-254.
10. Deconte, D., et al., *Ophthalmologic Impairment and Intellectual Disability in a Girl Presenting Kenny-Caffey Syndrome Type 2.* J Pediatr Genet, 2020. **9**(4): p. 263-269.
11. Cavole, T.R., et al., *Overlapping phenotype comprising Kenny-Caffey type 2 and Sanjad-Sakati syndromes: The first case report.* Am J Med Genet A, 2020. **182**(12): p. 3029-3034.
12. Wang, Y., et al., *Genetic Screening in a Large Chinese Cohort of Childhood Onset Hypoparathyroidism by Next-Generation Sequencing Combined with TBX1-MLPA.* J Bone Miner Res, 2019. **34**(12): p. 2254-2263.
13. Grosse, G., et al., *Targeted Resequencing of Putative Growth-Related Genes Using Whole Exome Sequencing in Patients with Severe Primary IGF-I Deficiency.* Horm Res Paediatr, 2017. **88**(6): p. 408-417.
14. Nikkel, S.M., et al., *Mother-to-daughter transmission of Kenny–Caffey syndrome associated with the recurrent, dominant FAM111A mutation p.Arg569His.* Clinical Genetics, 2014. **86**(4): p. 394-395.
15. Guo, M.H., et al., *Whole exome sequencing to identify genetic causes of short stature.* Horm Res Paediatr, 2014. **82**(1): p. 44-52.
16. Isojima, T., et al., *A recurrent de novo FAM111A mutation causes Kenny-Caffey syndrome type 2.* J Bone Miner Res, 2014. **29**(4): p. 992-8.
17. Unger, S., et al., *FAM111A mutations result in hypoparathyroidism and impaired skeletal development.* Am J Hum Genet, 2013. **92**(6): p. 990-5.
18. Fredriks, A.M., et al., *Nationwide age references for sitting height, leg length, and sitting height/height ratio, and their diagnostic value for disproportionate growth disorders.* Archives of Disease in Childhood, 2005. **90**(8): p. 807.
19. Kliegman, R.M., *Nelson textbook of pediatrics*. 21st edition. ed. 2019, Philadelphia, MO: Elsevier. pages cm.
20. Pagana, K.D., T.J. Pagana, and T.N. Pagana, *Mosby's diagnostic and laboratory test reference*. Sixteenth edition. ed. 2023, St. Louis, Missouri: Elsevier. xxiv, 1031 pages.
